# Higher beta-hydroxybutyrate ketone levels associated with a slower kidney function decline in ADPKD

**DOI:** 10.1093/ndt/gfad239

**Published:** 2023-11-16

**Authors:** Martine G E Knol, Thomas Bais, Paul Geertsema, Margery A Connelly, Stephan J L Bakker, Ron T Gansevoort, Maatje D A van Gastel, the DIPAK Consortium, J P H Drenth, J W de Fijter, D J M Peters, M Salih, E J Hoorn, T Nijenhuis, E Meijer

**Affiliations:** Department of Internal Medicine, Division of Nephrology, University of Groningen, University Medical Center Groningen, Groningen, The Netherlands; Department of Internal Medicine, Division of Nephrology, University of Groningen, University Medical Center Groningen, Groningen, The Netherlands; Department of Internal Medicine, Division of Nephrology, University of Groningen, University Medical Center Groningen, Groningen, The Netherlands; Labcorp, Morrisville, NC, USA; Department of Internal Medicine, Division of Nephrology, University of Groningen, University Medical Center Groningen, Groningen, The Netherlands; Department of Internal Medicine, Division of Nephrology, University of Groningen, University Medical Center Groningen, Groningen, The Netherlands; Department of Internal Medicine, Division of Nephrology, University of Groningen, University Medical Center Groningen, Groningen, The Netherlands

**Keywords:** ADPKD, beta-hydroxybutyrate, biomarker, ketone bodies, kidney function

## Abstract

**Background:**

Dysregulated energy metabolism is a recently discovered key feature of autosomal dominant polycystic kidney disease (ADPKD). Cystic cells depend on glucose and are poorly able to use other energy sources such as ketone bodies. Raising ketone body concentration reduced disease progression in animal models of polycystic kidney diseases. Therefore, we hypothesized that higher endogenous plasma beta-hydroxybutyrate (BHB) concentrations are associated with reduced disease progression in patients with ADPKD.

**Methods:**

We analyzed data from 670 patients with ADPKD participating in the Developing Intervention Strategies to Halt Progression of ADPKD (DIPAK) cohort, a multi-center prospective observational cohort study. BHB was measured at baseline using nuclear magnetic resonance spectroscopy. Participants were excluded if they had type 2 diabetes, were using disease-modifying drugs (e.g. tolvaptan, somatostatin analogs), were not fasting or had missing BHB levels, leaving 521 participants for the analyses. Linear regression analyses were used to study cross-sectional associations and linear mixed-effect modeling for longitudinal associations.

**Results:**

Of the participants, 61% were female, with an age of 47.3 ± 11.8 years, a height-adjusted total kidney volume (htTKV) of 834 [interquartile range (IQR) 495–1327] mL/m and an estimated glomerular filtration rate (eGFR) of 63.3 ± 28.9 mL/min/1.73 m^2^. The median concentration of BHB was 94 (IQR 68–147) µmol/L. Cross-sectionally, BHB was associated neither with eGFR nor with htTKV. Longitudinally, BHB was positively associated with eGFR slope {B = 0.35 mL/min/1.73 m^2^ [95% confidence interval (CI) 0.09 to 0.61], *P *= .007}, but not with kidney growth. After adjustment for potential confounders, every doubling in BHB concentration was associated with an improvement in the annual rate of eGFR by 0.33 mL/min/1.73 m^2^ (95% CI 0.09 to 0.57, *P *= .008).

**Conclusion:**

These observational analyses support the hypothesis that interventions that raise BHB concentration could reduce the rate of kidney function decline in patients with ADPKD.

## Graphical Abstract

**Graphical Abstract:**
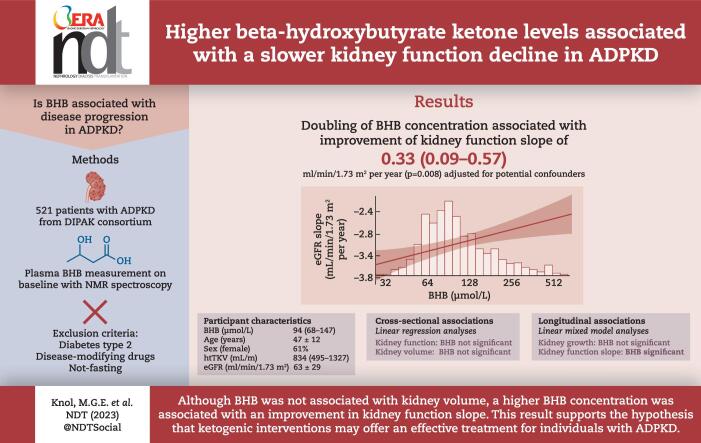

KEY LEARNING POINTS **What was known:**

• Autosomal dominant polycystic kidney disease (ADPKD) is characterized by a dysregulated metabolism. Intervening in this dysregulated metabolism by ketogenic interventions reduced the rate of disease progression in PKD animal models. The concentration of beta-hydroxybutyrate (BHB), a ketone body, increases during ketogenic interventions.

**This study adds:**

• The is the first large-scale epidemiological study investigating the association of BHB with kidney function decline in patients with ADPKD.
• It shows that even mildly elevated levels of BHB are associated with less kidney function decline in patients with ADPKD.

**Potential impact:**

• Emerging animal data show that ketogenic interventions are protective in ADPKD. This observational study in a large cohort of patients with ADPKD suggests that even a small increase in BHB may already have a positive effect on disease progression. These data warrant intervention trials.

## INTRODUCTION

Autosomal dominant polycystic kidney disease (ADPKD) is the most common genetic kidney disease, characterized by cyst growth in both kidneys. The progressive cyst growth leads to kidney function decline, and in most patients to end-stage kidney disease around the age of 60 years [1]. It is estimated that approximately 10% of all patients requiring dialysis or kidney transplantation have ADPKD as an underlying disease [2]. Currently, there is only one treatment option for reducing disease progression, tolvaptan, a vasopressin V2 receptor antagonist [3]. However, there is important residual disease progression when using this drug and tolvaptan use can lead to aquaretic side-effects such as polyuria, limiting its feasibility for many patients. Therefore, exploring other mechanisms that may be involved with disease progression is vital because of the potential to develop novel ways to treat ADPKD.

It was recently discovered that ADPKD cells have dysregulated glucose metabolism, suggesting that these cells mainly depend on glucose and aerobic glycolysis [4]. Therefore, they inefficiently use other energy metabolites, such as ketone bodies. Ketone bodies are formed by the liver when low glucose levels stimulate glucagon secretion and reduce insulin secretion, such as during fasting, a ketogenic diet or long-lasting exercise [5]. Increasing ketogenesis is thought to reduce disease progression by lowering glucose availability, which could reduce cyst growth, and by influencing signaling pathways. In experimental studies, ketogenic interventions successfully reduced disease progression in polycystic kidney disease animal models, possibly by lowering glucose availability in renal tubular cells and by reducing mammalian target of rapamycin (mTOR) signaling [6, 7]. mTOR is an important energy-sensing protein known to be upregulated in ADPKD and to stimulate cyst proliferation [8].

A ketogenic state enables the influx of free fatty acids into the liver, where the three main ketone bodies are synthesized: beta-hydroxybutyrate (BHB), acetoacetate and acetone [5]. BHB can suppress oxidative stress, diminish fibrosis and reduce inflammation, which could contribute to reducing disease progression in ADPKD [9–12]. BHB is the most abundant and stable ketone body and can be measured reliably using nuclear magnetic resonance (NMR) spectroscopy [13].

This study aims to investigate the association of BHB with disease severity and progression in a prospective cohort of patients with ADPKD, as it is unknown whether the findings in experimental studies have clinical relevance. We hypothesize that higher plasma BHB concentration is associated with less disease progression.

## MATERIALS AND METHODS

### Study design and study population

Data from the Developing Intervention Strategies to Halt Progression of ADPKD (DIPAK) observational cohort study was used for the current study. The DIPAK study was designed to investigate disease course in an unselected ADPKD patient population. Data were collected at four University Medical Centers in The Netherlands, in Groningen, Leiden, Nijmegen and Rotterdam. The cohort currently comprises 670 patients with ADPKD, with a current median follow-up of 4 years. Inclusion criteria were having ADPKD according to the modified Ravine criteria [14], age ≥18 years, and an estimated glomerular filtration rate (eGFR) ≥15 mL/min/1.73 m^2^.

For the present study participants with missing BHB levels (*n* = 38), who were not fasting or had unknown fasting status (*n* = 36), and subjects with extremely high or low BHB values [more than three times the standard deviation (SD) above or below the mean log-transformed variable, *n* = 4] were excluded. Moreover, patients were excluded who during follow-up started disease-modifying drugs (e.g. somatostatin analogs or vasopressin V2 receptor antagonists, *n* = 56), or who developed type 2 diabetes [hemoglobin A1c (HbA1c) >53 mmol/mol or fasting glucose >7 mmol/L, *n* = 15], because this may affect the rate of disease progression. In total, 521 participants were used for the current analyses.

The DIPAK observational study was approved by the Institutional Review Board of the University Medical Center Groningen (METC: 2013/040). It was conducted in adherence with the International Conference on Harmonization Good Clinical Practice guidelines. Written informed consent was obtained from all participants.

### Measurements

Before each study visit, patients were fasted from midnight onward. Blood and 24-h urine samples were collected during the visit for immediate, routine biochemistry and *PKD* mutation analysis and biobanked for later analyses. In addition, blood pressure, body weight and height were measured. Copeptin was measured as a surrogate for vasopressin using a sandwich immunoassay (Thermo Fisher Scientific, Berlin, Germany). Glucagon was measured using a sandwich enzyme-linked immunosorbent assay (ELISA; Mercodia A/S, Uppsala, Sweden), which was previously described in detail [15]. Serum creatinine was measured using an isotope dilution mass spectrometry traceable enzymatic method. The estimated glomerular filtration rate (eGFR) was calculated using the 2009 creatinine-based Chronic Kidney Disease Epidemiology Collaboration equation [16]. Details for the leucine measurement are reported elsewhere [17]. Body mass index (BMI) was calculated by dividing body weight in kilograms by squared body height in meters. Magnetic resonance imaging (MRI) was performed at baseline and every 3 years to assess total kidney volume (TKV), using a standardized protocol. Of the 521 included patients, there were 491 MRIs at baseline, 337 MRIs at Year 3 and 116 MRIs at Year 6 available for TKV measurement. TKV was measured by trained reviewers blinded to the patient's identity using semi-automated segmentation and a validated deep-learning approach [18, 19]. The height-adjusted TKV (htTKV) was calculated by dividing the TKV by height in meters. Patients were classified using the Mayo Clinic Classification based on their age-indexed htTKV value, as previously described [20]. Salt intake was calculated as 24-h sodium excretion, and protein intake was estimated by the method of Maroni *et al.* [21] by the following formula: protein intake = [urea excretion (mmol) × 0.028 + 0.031 × body weight (kg)] × 6.25.

### BHB measurement

Plasma BHB was measured using a Vantera Clinical Analyzer (Labcorp), a fully automated, high-throughput, 400 MHz proton (^1^H) NMR spectroscopy platform [22]. The NMR-based assay quantifies BHB with a mean inter-assay coefficient of variation (%CV) of 5.8 and a mean intra-assay %CV of 5.2. The long-term stability of BHB after 6 years is good (<1.5% bias) [13].

### Statistical analyses

We used R studio version 4.0.5 (Vienna, Austria) for the statistical analyses. A two-sided *P*-value <.05 was considered statistically significant.

We used the mean ± SD for normally distributed quantitative variables, the median and interquartile range (IQR) for non-normally distributed quantitative variables, and frequencies and percentages for categorical variables. All non-normally distributed variables were log2 transformed to meet the assumptions for linear regression and linear mixed model analyses. Extreme outliers were defined as three times above or below the SD of the (log-transformed) variable and were removed from the analyses [23]. In case of missing data, cases were removed listwise.

The baseline characteristics were stratified for sex-adjusted quartiles of BHB because BHB concentration is slightly elevated in women compared with men [24]. Group differences were determined using the one-way ANOVA for normally distributed variables, the Kruskal–Wallis test for non-normally distributed variables and the Chi-Square test for categorical variables.

To investigate what influences the plasma BHB concentration in the ADPKD population and could therefore be a possible confounder during subsequent analyses, we performed univariable linear regression analyses with variables of interest based on literature: sex, age, alcohol use, glucagon, glucose, copeptin, HbA1c, use of beta-blockers, systolic blood pressure (SBP) and BMI [5, 13, 24–31]. For continuous variables, the presence of non-linear relationships with BHB concentration was investigated by adding the squared variable to the regression analysis. In the next model, these associations were adjusted for age and sex. Subsequently, the stepwise backward model included all variables associated with BHB with a *P*-value of <.1 after adjustment for sex and age. Then the variables were excluded stepwise when the *P*-value was >.05. The variables sex and age remained in the final stepwise backward analysis, regardless of significance.

We examined whether BHB was cross-sectionally associated with kidney function and volume, both crude and after adjustment for potential confounders. We adjusted for confounders that are known to influence disease progression or can influence the BHB concentration [1, 27, 31]. eGFR and htTKV were the dependent variables, and BHB was the independent variable (crude model). Next, we adjusted for potential confounders: sex, age (Model 1); Model 1 plus plasma copeptin (Model 2); Model 2 plus SBP and *PKD* mutations (Model 3). The standard assumptions of linear regression (linearity, homoscedasticity and normal distribution of residuals) were evaluated for every model.

We investigated whether BHB was associated with kidney function over time and kidney growth using linear mixed-effects models for the longitudinal analyses. All participants included had at least two available eGFR and htTKV values. Slope and intercept were allowed to vary randomly with an unstructured covariance structure. Random effects were time and participants. Fixed effects were BHB levels, time and the interaction of BHB with time. We adjusted for the same potential confounders as in the cross-sectional analyses by adding the variables and their interaction with time. The added covariates were measured at baseline. For htTKV, the annual percentage growth in htTKV can be calculated by taking the antilog of the estimated derived.

We assessed the univariable associations between independent variables of interest and each of the dependent variables (eGFR baseline, htTKV baseline, eGFR slope and htTKV growth; Supplementary data, Table S1). Most variables were univariably significantly associated with the dependent variables. If they were not significantly associated, they were still added to the model because of literature evidence of influencing disease progression [1, 27, 31]. A correlation matrix and plot were made, which showed no multicollinearity (Supplementary data, Fig. S1).

Subgroup analyses were performed to study the association between BHB and eGFR slope to assess the robustness across different subgroups. The estimate of BHB was calculated using linear mixed-effects models in the different subgroups. The *P* for interaction was calculated by adding to the model an interaction term between the subgroup, BHB and time. The associations were adjusted for potential confounders, sex, age, copeptin, SBP and *PKD* mutations.

## RESULTS

### Baseline characteristics

Baseline characteristics of the overall cohort of 521 patients with ADPKD and according to sex-stratified quartiles of baseline BHB are shown in Table 1. Women had higher BHB levels than men [101 (IQR 72–161) µmol/L versus 87 (IQR 65–131) µmol/L, *P *= .004]. The median BHB concentration was 94 (IQR 68–147) µmol/L, and the mean eGFR was 63.3 ± 28.9 mL/min/1.73 m^2^. The mean age of the participants was 47.3 ± 11.8 years, and 61.0% were female. Most baseline characteristics—age, BMI, genetic mutation, blood pressure, eGFR, htTKV, glucose, HbA1c and glucagon—were not significantly different between the quartiles of BHB (all *P *> .05). There were significant differences between the quartiles in plasma leucine (*P *= .03) and plasma copeptin (*P *= .03). Copeptin had a U-shaped trend across the quartiles, with the second quartile having the lowest level [6.4 (IQR 4.0–11.9) pmol/L] and the fourth quartile having the highest level [9.0 (IQR 4.4–22.0) pmol/L].

**Table 1:** Baseline characteristics of the total cohort and according to sex-stratified quartiles of baseline BHB (µmol/L).

		**Sex-stratified quartiles of baseline BHB (µmol/L)**				
		**M <65.0**	**M 65.0–87.0**	**M 87.0–131.0**	**M >131.0**	
	**Total**	**F <72.0**	**F 72.0–101.0**	**F 101.0–161.0**	**F >161.0**	***P-*value**
Total (*n*)	521	134	129	131	127	
BHB (µmol/L)	94 (68–147)	58 (49–64)	82 (77–87)	114 (103–131)	213 (180–316)	<.001
Female [*n* (%)]	318 (61.0)	77 (60.6)	79 (62.2)	81 (60.0)	81 (61.4)	.9
Age (years)	47.3 ± 11.8	47.2 ± 11.2	45.9 ± 11.7	47.6 ± 11.5	48.6 ± 12.7	.33
Height (cm)	175 ± 12.5	176 ± 9.9	175 ± 18.1	175 ± 9.8	176 ± 10.2	.67
Weight (kg)	80.8 ± 16.7	81.6 ± 17.2	80.3 ± 17.3	82.3 ± 17.8	78.8 ± 15.2	.37
BMI (kg/m^2^)	26.1 ± 4.6	26.1 ± 4.3	26.1 ± 4.2	26.9 ± 5.4	25.5 ± 4.4	.13
Genetic mutation [*n* (%)]						.21
*PKD1* T	205 (39.3)	55 (41.0)	53 (41.1)	46 (35.1)	51 (40.2)	
*PKD1* NT	140 (26.9)	35 (26.1)	43 (33.3)	35 (26.7)	27 (21.3)	
*PKD2*	131 (25.1)	35 (26.1)	24 (18.6)	33 (25.2)	39 (30.7)	
Missing/other	45 (8.6)	9 (6.7)	9 (7.0)	17 (13.0)	11 (8.5)	
Blood pressure (mmHg)						
Systolic	130 ± 13.4	129 ± 12.7	132 ± 14.6	129 ± 12.2	129 ± 14.1	.42
Diastolic	79.6 ± 8.7	79.0 ± 8.6	80.6 ± 9.8	80.1 ± 8.4	78.9 ± 8.0	.33
Antihypertensive use [*n* (%)]	378 (72.6)	93 (69.4)	94 (72.9)	100 (76.3)	91 (71.7)	.65
Beta-blockers [*n* (%)]	106 (20.4)	26 (19.5)	31 (24.0)	25 (19.1)	24 (18.9)	.70
Albuminuria (mg/24 h)	30 (14–69)	30 (14–65)	27 (12–70)	36 (17–76)	28 (14–70)	.63
Creatinine (µmol/L)	107 (77–149)	110 (81–152)	98 (74–132)	106 (78–147)	113 (78–159)	.13
eGFR (mL/min/1.73 m^2^)	63.3 ± 28.9	61.2 ± 28.4	68.2 ± 29.0	63.5 ± 28.4	60.5 ± 29.6	.13
CKD stage baseline [*n* (%)]						.22
Stage 1 + 2	251 (48.3)	57 (43.2)	72 (55.8)	66 (51.9)	54 (42.5)	
Stage 3A + B	205 (39.4)	56 (42.4)	45 (34.9)	51 (38.9)	53 (41.7)	
Stage 4 + 5	63 (12.1)	19 (14.4)	12 (9.3)	12 (9.2)	20 (15.7)	
Copeptin (pmol/L)	7.9 (4.4–15.6)	7.6 (3.6–14.7)	6.4 (4.0–11.9)	8.9 (4.9–19.0)	9.0 (4.4–22.0)	.03
htTKV (mL/m)	834 (495–1327)	828 (517–1322)	803 (494–1232)	995 (483–1509)	782 (479–1259)	.41
Mayo Risk Classification [*n* (%)]						.39
Low risk	148 (28.5)	36 (26.9)	37 (28.7)	32 (25.4)	44 (34.1)	
Medium risk	182 (35.0)	51 (38.1)	51 (39.5)	43 (33.1)	38 (29.5)	
High risk	158 (30.4)	37 (27.6)	36 (27.9)	47 (36.2)	38 (29.5)	
Unknown	32 (6.2)	10 (7.5)	5 (3.9)	7 (5.4)	5 (4.1)	
Glucose (mmol/L)	5.2 ± 0.5	5.2 ± 0.5	5.2 ± 0.6	5.2 ± 0.6	5.1 ± 0.6	.34
HbA1c (mmol/mol)	36.1 ± 3.7	36.3 ± 3.2	36.0 ± 3.9	36.3 ± 3.8	35.7 ± 4.1	.51
Glucagon (pmol/L)	5.0 (3.4–7.3)	4.8 (3.4–6.7)	4.6 (3.0–6.6)	5.0 (3.5–7.9)	5.6 (3.7–8.1)	.12
Leucine (µmol/L)	133 ± 29	127 ± 26	137 ± 32	136 ± 29	131 ± 29	.03
Alcohol use (yes) [*n* (%)]	410 (83.5)	97 (78.9)	105 (85.4)	102 (81.0)	106 (83.5)	.14

Normally distributed data are presented as mean ± SD, non-normally distributed as median (IQR) and categorical variables are presented as frequencies and percentages. *P-*values for differences between groups were tested with one-way ANOVA for normally distributed data, Kruskal–Wallis test for non-normally distributed data and Pearson Chi-Square for categorical data.

Distribution of Mayo Risk Classification: low risk (class 1A, 1B and 2); medium risk (class 1C); and high risk (class 1D and 1E).

M, male; F, female; PKD, polycystic kidney disease; T, truncating; NT, non-truncating; CKD, chronic kidney disease.

### Determinants of BHB concentration

In univariable analyses, sex and glucose were significantly associated with BHB concentration (Table 2). Sex, glucagon, glucose, copeptin and HbA1c were associated with BHB concentration after adjustment for sex and age. In the stepwise backward analysis, sex {B = 1.205 [95% confidence interval (CI) 1.074–1.352], *P *= .001}, age [B = 1.005 (95% CI 1.001–1.009), *P *= .03] and glucagon [B = 1.020 (95% CI 1.005–1.035), *P *= .01] remained linearly associated with BHB, whereas glucose was nonlinearly significantly associated with BHB (*P *= .02). The relationship between BHB and glucose is depicted in Supplementary data, Fig. S2.

**Table 2:** Associations of possible determinants of baseline log_2_(BHB) concentration.

	**Univariable analyses**		**Sex and age-adjusted**		**Stepwise backward analysis**	
	**B (95% CI)**	***P-*value**	**B (95% CI)**	***P-*value**	**B (95% CI)**	***P-*value**
Sex (female)	1.153 (1.037–1.281)	.008	1.169 (1.051–1.299)	.004	1.205 (1.074–1.352)	.001
Age (years)	1.003 (0.999–1.008)	.13	1.004 (1.000–1.009)	.06	1.005 (1.001–1.009)	.03
Alcohol use (ref: no)	1.103 (0.957–1.270)	.17	1.142 (0.990–1.317)	.07		
Glucagon (pmol/L)	1.011 (0.997–1.025)	.12	1.019 (1.004–1.034)	.01	1.020 (1.005–1.035)	.01
Glucose (mmol/L)	0.199 (0.059–0.676)	.01	0.207 (0.061–0.696)	.01	0.221 (0.064–0.755)	.02
Glucose^2^	1.154 (1.030–1.293)	.01	1.151 (1.028–1.288)	.01	1.143 (1.019–1.281)	.02
Log_2_(copeptin)	1.036 (0.994–1.080)	.09	1.064 (1.016–1.114)	.008		
HbA1c (mmol/mol)	0.988 (0.975–1.002)	.09	0.984 (0.970–0.999)	.03		
Beta-blockers (yes)	0.923 (0.812–1.049)	.22	0.893 (0.784–1.017)	.09		
SBP (mmHg)	0.998 (0.994–1.002)	.26	0.998 (0.994–1.002)	.25		
BMI (kg/m^2^)	0.993 (0.982–1.004)	.22	0.993 (0.982–1.004)	.23		

Estimates, CI and *P*-values were calculated using linear regression analysis. The dependent variable is log_2_(BHB). Independent variables are sex, age, alcohol use, glucagon, glucose, copeptin, HbA1c, beta-blockers, SBP and BMI. The estimates are back-log transformed, for the categorical variables indicating the fold change in BHB for the specified category compared with the reference category. For the continuous variables, the estimate represents the fold-change per one-unit increase in the continuous variable. When the independent variable is log_2_-transformed, a doubling (one unit increase) of the independent variable corresponds to the fold change according to the estimate.

B, estimate.

### Cross-sectional analyses

Multivariable linear regression analyses were used to assess the cross-sectional associations of BHB with eGFR and htTKV (Table 3). BHB was not associated with eGFR at baseline [B = **–**1.07 (95% CI –3.95 to 1.81), *P *= .47]. After additional adjustment for potential confounders sex, age and copeptin, the association of BHB with eGFR was close to significance [B = 1.52 (95% CI –0.21 to 3.24), *P *= .08]. Nevertheless, in the final model, after additional adjustment for SBP and *PKD* mutations, BHB remained not significantly associated with eGFR [B = 1.29 (95% CI –0.38 to 2.96), *P *= .13].

**Table 3:** Cross-sectional associations of BHB with eGFR and htTKV on baseline using linear regression analyses.

	**Crude**		**Model 1**		**Model 2**		**Model 3**	
**Variables**	**B (95% CI)**	***P-*value**	**B (95% CI)**	***P-*value**	**B (95% CI)**	***P-*value**	**B (95% CI)**	***P-*value**
eGFR	R^2 ^= 0.001; *N* = 520		R^2 ^= 0.46; *N* = 520		R^2 ^= 0.66; *N* = 513		R^2 ^= 0.68; *N* = 513	
Log_2_(BHB)	–1.07 (–3.95 to 1.81)	.47	–0.01 (–2.14 to 2.13)	.9	1.52 (–0.21 to 3.24)	.08	1.29 (–0.38 to 2.96)	.13
Sex (female)			6.08 (2.26 to 9.89)	.002	–5.12 (–8.44 to –1.8)	.003	–4.39 (–7.63 to –1.14)	.008
Age (years)			–1.62 (–1.78 to –1.46)	<.001	–1.36 (–1.49 to –1.23)	<.001	–1.52 (–1.66 to –1.38)	<.001
Log_2_(copeptin)					–11.54 (–12.87 to –10.21)	<.001	–10.90 (–12.20 to –9.59)	<.001
SBP							0.04 (–0.07 to 0.14)	.52
*PKD2* (ref)^a^								
*PKD1* NT							–6.00 (–10.07 to –1.94)	.004
*PKD1* T							–10.48 (–14.43 to –6.53)	<.001
Other/missing							4.46 (–1.43 to 10.34)	.31
htTKV	R^2 ^= 0.002; *N* = 489		R^2 ^= 0.15; *N* = 489		R^2 ^= 0.26; *N* = 482		R^2 ^= 0.29; *N* = 482	
Log_2_(BHB)	0.961 (0.895 to 1.032)	.27	0.977 (0.915 to 1.044)	.50	0.950 (0.893 to 1.011)	.11	0.952 (0.895 to 1.012)	.12
Sex (female)			0.671 (0.596 to 0.755)	<.001	0.822 (0.728 to 0.927)	<.001	0.818 (0.726 to 0.922)	<.001
Age (years)			1.013 (1.008 to 1.018)	<.001	1.009 (1.004 to 1.013)	<.001	1.010 (1.005 to 1.015)	<.001
Log_2_(copeptin)					1.229 (1.171 to 1.291)	<.001	1.219 (1.161 to 1.280)	<.001
SBP							1.003 (0.999 to 1.007)	.14
*PKD2* (ref)^a^								
*PKD1* NT							0.989 (0.852 to 1.148)	.9
*PKD1* T							1.146 (0.989 to 1.327)	.07
Other/missing							0.781 (0.635 to 0.959)	.02

Estimates and *P*-values were calculated using multivariable regression analyses. The dependent variables are eGFR (mL/min/1.73m^2^) and log_2_(htTKV) (mL/m) at baseline. The independent variables are baseline log_2_(BHB) (crude), adjusted for sex, age (Model 1), additionally adjusted for log_2_(copeptin) (Model 2) and additionally adjusted for SBP and *PKD* mutations (Model 3). The estimates for dependent variable htTKV are back-log transformed. For categorical variables, estimates show fold changes compared with the reference category. For continuous variables, the estimate indicates the fold change per one-unit increase. When the independent variable is log_2_-transformed, a doubling (one unit increase) of the independent variable corresponds to the fold change according to the estimate.

^a^*PKD* mutation was used as dummy variable with *PKD2* as reference group.

B, estimate; *N*, number; PKD, polycystic kidney disease; NT, non-truncating; T, truncating.

BHB did not associate with htTKV at baseline [B = 0.961 (95% CI 0.895 to 1.032), *P *= .27]. Also, after adjustment for potential confounders and known determinants of disease progression, BHB remained not associated with htTKV [B = 0.952 (95% CI 0.895 to 1.012), *P *= .12].

As a sensitivity analysis, participants with type 2 diabetes were added to the cross-sectional analysis (*n* = 15). The association between BHB and eGFR at baseline did not change after adding these participants [crude, B = –1.23 (–4.09 to 1.64), *P *= .40; and Model 3, B = 1.47 (–0.20 to 3.14), *P *= .08]. Similar results were obtained for the association with htTKV in the final model including participants with type 2 diabetes [B = 0.952 (95% CI 0.985 to 1.012), *P *= .11].

### Longitudinal analyses

Linear mixed model analyses were used to assess longitudinal associations. A total of 489 participants with at least two eGFR measurements were included in these analyses. The median follow-up time was 3.95 (IQR 2.99–6.00) years. The median change in eGFR was –2.97 (95% CI –3.18 to –2.76) mL/min/1.73 m^2^ per year. BHB was positively associated with the eGFR slope [B = 0.35 (95% CI 0.09 to 0.61), *P *= .005] (Table 4). The model estimated that each doubling of BHB was associated with an improvement in the rate of eGFR decline by 0.35 mL/min/1.73 m^2^ per year. In all subsequent multivariable models, BHB remained positively associated with eGFR. After adding copeptin, the association became stronger [B = 0.40 (95% CI 0.15 to 0.65), *P *= .002]. In the final model, after adjustment for sex, age, copeptin, SBP and *PKD* mutations, each doubling in BHB concentration was associated with an improvement in the eGFR slope of 0.33 (95% CI 0.09 to 0.57, *P *= .008) mL/min/1.73 m^2^ per year. To assess whether the association between BHB and the eGFR slope was independent of the htTKV, we added the htTKV on baseline to the final model. BHB remained significantly associated with the eGFR slope after this additional adjustment [B = 0.29 (95% CI 0.05 to 0.53), *P *= .02].

**Table 4:** Longitudinal associations of BHB between eGFR/year and htTKV/year using linear mixed model analyses.

	**Crude**		**Model 1**		**Model 2**		**Model 3**	
**Variables**	**Est. (95% CI)**	***P-*value**	**Est. (95% CI)**	***P-*value**	**Est. (95% CI)**	***P-*value**	**Est. (95% CI)**	***P-*value**
eGFR slope	R^2 ^= 0.04, *N* = 489		R^2 ^= 0.43, *N* = 489		R^2 ^= 0.63, *N* = 482		R^2 ^= 0.67, *N* = 482	
Log_2_(BHB)	0.35 (0.09 to 0.61)	.007	0.30 (0.05 to 0.56)	.02	0.40 (0.15 to 0.65)	.002	0.33 (0.09 to 0.57)	.008
Sex (female)			0.59 (0.14 to 1.04)	.005	–0.01 (–0.48 to 0.45)	.9	–0.04 (–0.49 to 0.41)	.85
Age (years)			0.00 (–0.02 to 0.02)	.9	0.02 (0.00 to 0.04)	.048	0.01 (–0.01 to 0.03)	.45
Log_2_(copeptin)					–0.65 (–0.85 to –0.45)	<.001	–0.57 (–0.77 to –0.38)	<.001
SBP							–0.03 (–0.05 to –0.02)	<.001
*PKD2* (ref)*								
*PKD1* NT							–0.89 (–1.45 to –0.32)	0.002
*PKD1* T							–1.19 (–1.75 to –0.63)	<.001
Other/missing							0.06 (–0.74 to 0.85)	.9
htTKV growth/year	R^2 ^= 0.03, *N* = 348		R^2 ^= 0.16, *N* = 348		R^2 ^= 0.22, *N* = 344		R^2 ^= 0.26, *N* = 344	
Log_2_(BHB)	1.000 (0.995 to 1.005)	.9	1.001 (0.997 to 1.006)	.55	1.001 (0.996 to 1.006)	.74	1.001 (0.996 to 1.006)	.67
Sex (female)			0.983 (0.974 to 0.991)	<.001	0.987 (0.978 to 0.996)	.004	0.988 (0.979 to 0.996)	.006
Age (years)			1.000 (0.9995 to 1.00)	.48	1.000 (0.999 to 1.000)	.12	1.000 (0.999 to 1.000)	.20
Log_2_(copeptin)					1.006 (1.002 to 1.010)	.002	1.006 (1.002 to 1.01)	.003
SBP							1.000 (1.000 to 1.001)	.13
*PKD2* (ref)*								
*PKD1* NT							0.997 (0.986 to 1.008)	.63
*PKD1* T							1.001 (0.990 to 1.012)	.89
Other/missing							0.975 (0.960 to 0.989)	.001

The estimates and *P*-values were calculated using linear mixed model analyses. The estimates are the variables and their interaction with time which is the effect of the variables on eGFR slope (mL/min/1.73 m^2^ per year) (top part of the table) or htTKV growth per year (bottom part of the table).

The independent variables are baseline log_2_(BHB) (crude), adjusted for sex, age (Model 1), additionally adjusted for log_2_(copeptin) (Model 2) and additionally adjusted for SBP and *PKD* mutations (Model 3). The estimates for dependent variable htTKV growth are back-log transformed. For categorical variables, estimates show fold changes compared with the reference category. For continuous variables, the estimate indicates the fold change per one-unit increase. When the independent variable is log_2_-transformed, a doubling (one unit increase) of the independent variable corresponds to the fold change according to the estimate. The fold change can be calculated to percentage difference = (fold change – 1)*100%.

*PKD mutation was used as dummy variable with *PKD2* as reference group.

Est., estimate; PKD, polycystic kidney disease; NT, non-truncating; T, truncating.

**Figure 1:**
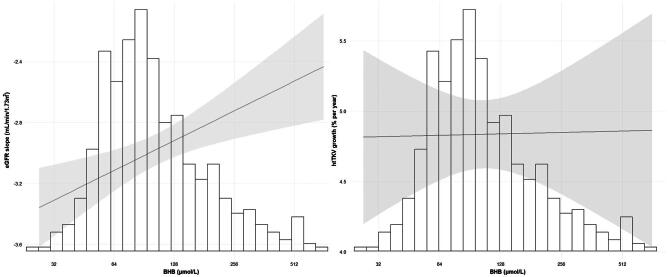
The distribution of BHB (µmol/L) and the association of BHB with the eGFR slope (left panel) and htTKV growth (right panel). The line estimates the influence of BHB on the eGFR slope and htTKV growth adjusted for sex, age, copeptin, SBP and *PKD* mutations. The shaded area is the 95% CI of the estimate. The *x*-axes represent the back-log transformed BHB values and its corresponding histogram. The *y*-axis on the left panel represents the eGFR slope (mL/min/1.73 m^2^) and on the right panel the htTKV growth (% per year).

A total of 348 participants had at least two available TKV measurements. The median growth rate of htTKV was 4.84 (95% CI 4.42 to 5.23) % per year. BHB was not associated with a change in htTKV over time [B = 1.000 (95% CI 0.995 to 1.005), *P *= .9] (Table 4). BHB remained not significantly associated with htTKV over time in all multivariable models. The relation between BHB and eGFR slope and htTKV growth is shown in Fig. 1.

As sensitivity analyses, participants with type 2 diabetes (*n* = 15) were added to the longitudinal analysis. The results remained essentially similar: each doubling in BHB concentration remained associated with an improvement in eGFR slope by 0.34 (95% CI 0.10 to 0.57, *P *= .006) mL/min/1.73 m^2^ per year in the final model, whereas the association of BHB with htTKV over time remained not significant [final model, B = 1.001 (95% CI 0.996 to 1.006), *P *= .75]. In addition, the association between BHB and the eGFR slope remained significant after additional adjustment for the BHB determinants age, sex, glucagon and glucose [B = 0.33 (95% CI 0.08 to 0.58), *P *= .01]. Since glucose was nonlinearly significantly associated with BHB, and is an easier to measure biomarker, we assessed the association of glucose in quartiles and continuously on the eGFR slope. Glucose in quartiles and continuously was not significantly associated with the eGFR slope crude, nor after adjustment for sex, age, copeptin, SBP and *PKD* mutations (*P *> .50). To assess the effect of dietary intake of salt and proteins, we additionally adjusted for these potential confounders in the final model. BHB remained significantly associated with the eGFR slope [B = 0.28 (95% CI 0.05 to 0.51), *P *= .02]. The robustness of the association between BHB and eGFR slope across different subgroups was assessed as shown in Supplementary data, Table S2. The BHB estimate remained positively associated with an improvement in eGFR across the different subgroups: sex, age, baseline eGFR, baseline htTKV and copeptin. Although not all estimates were significant due to loss of power, the overall trend was a positive association between BHB and eGFR slope across the subgroups. There were no significant interactions between the subgroups and BHB. To assess if the inclusion of the outliers altered the results, these participants (*n* = 4) were added to the analyses. BHB remained positively significantly associated with the eGFR slope [B = 0.27 (95% CI 0.05 to 0.50), *P *= .02].

## DISCUSSION

In the present study, we found that in a general ADPKD population cohort, higher concentrations of the ketone body BHB were associated with less kidney function decline. While we did not observe an association between BHB and changes in htTKV over time, the favorable relationship between BHB and the eGFR slope holds great promise. The finding that BHB is associated with eGFR but not htTKV is noteworthy, since this study was predicated on animal and *in vitro* data that predicted that ketogenesis would inhibit kidney cyst (and hence volume) growth. Instead, we appear to have found a biomarker of GFR decline that is independent of cyst growth. Hypothetically, this could be because the possible protective effect of BHB on kidney function might be an ADPKD-independent effect, since in other preclinical studies raising BHB concentration ameliorated the rate of kidney disease progression [11, 32, 33]. These results provide support for the hypothesis that ketogenic interventions may offer an effective treatment approach for individuals with ADPKD.

The positive effect of BHB on the eGFR slope is in line with data from preclinical studies, where ketogenic interventions successfully reduced disease progression [6, 7]. Only a few clinical studies are available on the effect of ketogenic interventions in ADPKD, including a retrospective case series study and two interventional studies. The retrospective study included 131 patients with ADPKD who followed either a ketogenic diet, time-restricted feeding or caloric restriction [34]. The results indicated an increase in eGFR of 3.6 mL/min/1.73 m^2^ after a median treatment duration of 6 months. However, the eGFR data were self-reported and not collected in a standardized manner, limiting their reliability [34]. Recently, two ketogenic intervention trials were conducted in patients with ADPKD (NCT04680780 and NCT04472624). The RESET-PKD trial was a pilot study that demonstrated that 90% of the participants could reach an effective level of ketogenesis (defined as a BHB >0.8 mmol/L) [35]. The second trial, the KETO-ADPKD study, included 63 patients and lasted 3 months. Preliminary results showed a significant increase in eGFR and a close to significant reduction in htTKV among participants on the ketogenic diet compared with the control group [36]. These interventional studies indicate that reaching an adequate level of ketogenesis is feasible and might positively impact disease progression in patients with ADPKD, even after a short treatment period. Nevertheless, additional research is needed to confirm the potential protective effects of ketogenic interventions in ADPKD during a longer treatment period. In addition to the previous investigations, our study demonstrates that mildly elevated BHB concentrations, even in a non-ketogenic range, are associated with an improvement of the eGFR slope by 0.33 mL/min/1.73 m^2^ per year after adjustment for potential confounders and known determinants of disease progression.

The alleged protective effects of BHB have been attributed to several factors. ADPKD cells have a dysregulated metabolism, shifting from oxidative phosphorylation to aerobic glycolysis, similar to the Warburg effect in cancer cells [4]. This alteration makes the cells dependent on glucose for growth and survival, and it leads to changes in multiple energy sensors in the cell, such as the upregulation of mTOR and the downregulation of AMP-activated kinase (AMPK) [37–39]. These alterations are known to influence disease progression in ADPKD negatively. Ketogenic interventions have been shown to reduce mTOR and increase AMPK signaling in ADPKD [6]. Besides changing the signaling of these energy sensors, BHB can reduce inflammation, oxidative stress and fibrosis by blocking the inflammasome and modulating gene expression via histone deacetylases [9, 10].

Interestingly, our data indicate that there might be a relation between the vasopressin axis and ketogenesis. Copeptin, a surrogate marker of vasopressin, is known to be increased in ADPKD and to influence disease progression negatively [40]. Vasopressin may also have an impact on ketogenesis and glucagon concentration [31, 41, 42]. Preclinical studies in rats showed mixed effects: low vasopressin reduced ketone bodies, and high vasopressin increased glucagon, stimulating ketogenesis [31, 41]. This phenomenon was also observed in healthy volunteers, in whom a vasopressin infusion did not change BHB levels, while glucagon levels were increased [43]. In the present study, copeptin was associated with higher BHB levels, adjusted for age and sex, but this association lost significance in the stepwise backward analysis, whereas glucagon stayed positively associated. Therefore, how strong the effect of vasopressin on ketogenesis is remains to be elucidated. Of note, it may be clinically relevant when vasopressin would influence ketogenesis in patients with ADPKD. Vasopressin concentration is known to be elevated in this population and, according to the literature, may have a negative impact on ketogenesis. At the same time, we also show that increased ketone body levels are associated with better kidney function outcome. These findings may thus suggest a novel mechanism by which vasopressin could work deleteriously in ADPKD.

The strengths of the study are the well-phenotyped and large cohort of patients with ADPKD, the sensitive and specific measurement of BHB, and the serial measurements of eGFR and htTKV as measures of disease severity and progression. Limitations are, firstly, that BHB was only measured at baseline. Therefore, we do not know the levels of BHB during follow-up and whether changes in BHB influenced the eGFR slope over time. Secondly, no patients had BHB levels in the ketogenic range (>800 µmol/L). We therefore cannot determine the effect of being in a ketogenic state on disease progression in ADPKD. Nevertheless, our findings show that even mildly increased levels of BHB at baseline are associated with an improvement in the eGFR slope. The association of doubling the BHB concentration with an improvement of the kidney function of 0.33 mL/min/1.73 m^2^ per year would imply an improvement of around 3–4 mL/min/1.73 m^2^ after 10 years of follow-up. Potentially, during a ketogenic diet, the BHB levels could increase 4- to 8-fold, which could translate to an improvement of eGFR around 9–12 mL/min/1.73m^2^ after 10 years of follow-up, which would be clinically significant. Lastly, as in all observational studies, we cannot infer causality from our results, and there might be residual confounding.

In conclusion, this study is the first to find that higher plasma ketone body beta-hydroxybutyrate concentrations are associated with less kidney function decline in a large observational cohort of patients with ADPKD. Our observational data support the hypothesis that raising beta-hydroxybutyrate levels by diet or drugs could reduce the rate of disease progression in patients with ADPKD.

## Supplementary Material

gfad239_Supplemental_File

## Data Availability

Data cannot be shared for legal reasons. Participating subjects signed in their informed consent form that their data will only be used by investigators of the DIPAK Consortium and will not be handed to third parties. However, external investigators can submit a research proposal to the DIPAK Steering Committee. If this body agrees with the proposal, the proposed analyses will be carried out by the epidemiologists working for the consortium. The results will be sent to these investigators and may be used for publication.

## References

[1] Cornec-Le Gall E, Alam A, Perrone RD. Autosomal dominant polycystic kidney disease. Lancet 2019;393:919–35. 10.1016/S0140-6736(18)32782-X 30819518

[2] Spithoven EM, Kramer A, Meijer E et al. Analysis of data from the ERA-EDTA Registry indicates that conventional treatments for chronic kidney disease do not reduce the need for renal replacement therapy in autosomal dominant polycystic kidney disease. Kidney Int 2014;86:1244–52. 10.1038/ki.2014.120 24827775

[3] Bais T, Gansevoort RT, Meijer E. Drugs in clinical development to treat autosomal dominant polycystic kidney disease. Drugs 2022;82:1095–115. 10.1007/s40265-022-01745-9 35852784 PMC9329410

[4] Rowe I, Chiaravalli M, Mannella V et al. Defective glucose metabolism in polycystic kidney disease identifies a new therapeutic strategy. Nat Med 2013;19:488–93. 10.1038/nm.3092 23524344 PMC4944011

[5] Puchalska P, Crawford PA. Multi-dimensional roles of ketone bodies in fuel metabolism, signaling, and therapeutics. Cell Metab 2017;25:262–84. 10.1016/j.cmet.2016.12.022 28178565 PMC5313038

[6] Torres JA, Kruger SL, Broderick C et al. Ketosis ameliorates renal cyst growth in polycystic kidney disease. Cell Metab 2019;30:1007–23. 10.1016/j.cmet.2019.09.012 31631001 PMC6904245

[7] Warner G, Hein KZ, Nin V et al. Food restriction ameliorates the development of polycystic kidney disease. J Am Soc Nephrol 2016;27:1437–47. 10.1681/ASN.2015020132 26538633 PMC4849816

[8] Shillingford JM, Murcia NS, Larson CH et al. The mTOR pathway is regulated by polycystin-1, and its inhibition reverses renal cystogenesis in polycystic kidney disease. Proc Natl Acad Sci USA 2006;103:5466–71. 10.1073/pnas.0509694103 16567633 PMC1459378

[9] Youm YH, Nguyen KY, Grant RW et al. The ketone metabolite β-hydroxybutyrate blocks NLRP3 inflammasome-mediated inflammatory disease. Nat Med 2015;21:263–9. 10.1038/nm.3804 25686106 PMC4352123

[10] Shimazu T, Hirschey M, Newman J et al. Suppression of oxidative stress by β-hydroxybutyrate, an endogenous histone deacetylase inhibitor. Science 2013;339:211–4. 10.1126/science.1227166 23223453 PMC3735349

[11] Rojas-Morales P, Pedraza-Chaverri J, Tapia E. Ketone bodies for kidney injury and disease. Adv Redox Res 2021;2:100009. 10.1016/j.arres.2021.100009

[12] Izuta Y, Imada T, Hisamura R et al. Ketone body 3-hydroxybutyrate mimics calorie restriction via the Nrf2 activator, fumarate, in the retina. Aging Cell 2018;17:e12699. 10.1111/acel.12699 29119686 PMC5770878

[13] Garcia E, Shalaurova I, Matyus SP et al. Ketone bodies are mildly elevated in subjects with type 2 diabetes mellitus and are inversely associated with insulin resistance as measured by the lipoprotein insulin resistance index. J Clin Med 2020;9:321. 10.3390/jcm9020321 31979327 PMC7074331

[14] Pei Y, Obaji J, Dupuis A et al. Unified criteria for ultrasonographic diagnosis of ADPKD. J Am Soc Nephrol 2009;20:205–12. 10.1681/ASN.2008050507 18945943 PMC2615723

[15] Knol MGE, Kramers BJ, Gansevoort RT et al. The association of glucagon with disease severity and progression in patients with autosomal dominant polycystic kidney disease: an observational cohort study. Clin Kidney J 2021;14:2582–90. 10.1093/ckj/sfab112 34950469 PMC8690142

[16] Levey AS, Stevens LA, Schmid CH et al. A new equation to estimate glomerular filtration rate. Ann Intern Med 2009;150:604. 10.7326/0003-4819-150-9-200905050-00006 19414839 PMC2763564

[17] Flores-Guerrero JL, Groothof D, Connelly MA et al. Concentration of branched-chain amino acids is a strong risk marker for incident hypertension. Hypertension 2019;74:1428–35. 10.1161/HYPERTENSIONAHA.119.13735 31587574

[18] van Gastel MDA, Edwards ME, Torres VE et al. Automatic measurement of kidney and liver volumes from MR Images of patients affected by autosomal dominant polycystic kidney disease. J Am Soc Nephrol 2019;30:1514–22. 10.1681/ASN.2018090902 31270136 PMC6683702

[19] Kline TL, Korfiatis P, Edwards ME et al. Performance of an artificial multi-observer deep neural network for fully automated segmentation of polycystic kidneys. J Digit Imaging 2017;30:442–8. 10.1007/s10278-017-9978-1 28550374 PMC5537093

[20] Irazabal Mv, Abebe KZ, Bae KT et al. Prognostic enrichment design in clinical trials for autosomal dominant polycystic kidney disease: the HALT-PKD clinical trial. Nephrol Dial Transplant 2017;32:1857–65. 27484667 10.1093/ndt/gfw294 PMC5837227

[21] Maroni BJ, Steinman TI, Mitch WE. A method for estimating nitrogen intake of patients with chronic renal failure. Kidney Int 1985;27:58–65. 10.1038/ki.1985.10 3981873

[22] Matyus SP, Braun PJ, Wolak-Dinsmore J et al. NMR measurement of LDL particle number using the Vantera® Clinical Analyzer. Clin Biochem 2014;47:203–10. 10.1016/j.clinbiochem.2014.07.015 25079243

[23] Osborne JW, Overbay A. The power of outliers (and why researchers should ALWAYS check for them). PARE 2004;9:6.

[24] Halkes CJM, van Dijk H, Verseyden C et al. Gender differences in postprandial ketone bodies in normolipidemic subjects and in untreated patients with familial combined hyperlipidemia. Arterioscler Thromb Vasc Biol 2003;23:1875–80. 10.1161/01.ATV.0000092326.00725.ED 12933534

[25] Merimee TJ, Misbin RI, Pulkkinen AJ. Sex variations in free fatty acids and ketones during fasting: evidence for a role of glucagon. J Clin Endocrinol Metab 1978;46:414–9. 10.1210/jcem-46-3-414 752030

[26] Eap B, Nomura M, Panda O et al. Ketone body metabolism declines with age in mice in a sex-dependent manner. biorxiv 2022;10.05.511032; not peer reviewed.

[27] Chakraborty S, Galla S, Cheng X et al. Salt-responsive metabolite, β-hydroxybutyrate, attenuates hypertension. Cell Rep 2018;25:677–89.e4. 10.1016/j.celrep.2018.09.058 30332647 PMC6542293

[28] Lefèvre A, Adler H, Lieber CS. Effect of ethanol on ketone metabolism. J Clin Invest 1970;49:1775–82. 10.1172/JCI106395 5456793 PMC322666

[29] Higashino-Matsui Y, Shirato K, Suzuki Y et al. Age-related effects of fasting on ketone body production during lipolysis in rats. Environ Health Prev Med 2012;17:157–63. 10.1007/s12199-011-0231-0 21850422 PMC3342632

[30] Vice E, Privette JD, Hickner RC et al. Ketone body metabolism in lean and obese women. Metabolism 2005;54:1542–5. 10.1016/j.metabol.2005.05.023 16253646

[31] Harano A, Hidaka H, Kojima H et al. Suppressive effect of vasopressin on ketosis in diabetic rats. Horm and Metab Res 1992;24:5–9. 10.1055/s-2007-1003241 1612560

[32] Tajima T, Yoshifuji A, Matsui A et al. β-hydroxybutyrate attenuates renal ischemia-reperfusion injury through its anti-pyroptotic effects. Kidney Int 2019;95:1120–37. 10.1016/j.kint.2018.11.034 30826015

[33] Wei T, Tian W, Liu F et al. Protective effects of exogenous β-hydroxybutyrate on paraquat toxicity in rat kidney. Biochem Biophys Res Commun 2014;447:666–71. 10.1016/j.bbrc.2014.04.074 24755084

[34] Strubl S, Oehm S, Torres JA et al. Ketogenic dietary interventions in autosomal dominant polycystic kidney disease—a retrospective case series study: first insights into feasibility, safety and effects. Clin Kidney J 2022;15:1079–92. 10.1093/ckj/sfab162 35664270 PMC9155228

[35] Oehm S, Steinke K, Schmidt J et al. RESET-PKD: a pilot trial on short-term ketogenic interventions in autosomal dominant polycystic kidney disease. Nephrol Dial Transplant 2023;38:1623–35. 10.1093/ndt/gfac311 36423335 PMC10435930

[36] Cukoski S, Lindemann C, Brecht T et al. KETO-ADPKD: a randomized, controlled trial on ketogenic dietary interventions in autosomal dominant polycystic kidney disease. Paper presented at: American Society of Nephrology Kidney Week, 2022, Orlando, FL, USA.

[37] Shillingford JM, Piontek KB, Germino GG et al. Rapamycin ameliorates PKD resulting from conditional inactivation of Pkd1. J Am Soc Nephrol 2010;21:489–97. 10.1681/ASN.2009040421 20075061 PMC2831854

[38] Podrini C, Cassina L, Boletta A. Metabolic reprogramming and the role of mitochondria in polycystic kidney disease. Cell Signal 2020;67:109495. 10.1016/j.cellsig.2019.109495 31816397

[39] Takiar V, Nishio S, Seo-Mayer P et al. Activating AMP-activated protein kinase (AMPK) slows renal cystogenesis. Proc Natl Acad Sci USA 2011;108:2462–7. 10.1073/pnas.1011498108 21262823 PMC3038735

[40] Boertien WE, Meijer E, Li J et al. Relationship of copeptin, a surrogate marker for arginine vasopressin, with change in total kidney volume and GFR decline in autosomal dominant polycystic kidney disease: results from the CRISP cohort. Am J Kidney Dis 2013;61:420–9. 10.1053/j.ajkd.2012.08.038 23089511 PMC3574620

[41] Rofe AM, Williamson DH. Metabolic effects of vasopressin infusion in the starved rat. Reversal of ketonaemia. Biochem J 1983;212:231–9. 10.1042/bj2120231 6135420 PMC1152034

[42] Kim A, Knudsen JG, Madara JC et al. Arginine-vasopressin mediates counter-regulatory glucagon release and is diminished in type 1 diabetes. eLife 2021;10:e72919. 10.7554/eLife.72919 34787082 PMC8654374

[43] Spruce BA, Mcculloch AJ, Burd J et al. The effect of vasopressin infusion on glucose metabolism in man. Clin Endocrinol (Oxf) 1985;22:463–8. 10.1111/j.1365-2265.1985.tb00145.x 3886209

