# Supplementary material for: Higher beta-hydroxybutyrate ketone levels associated with a slower kidney function decline in ADPKD
Source: Nephrol Dial Transplant. 2023 Nov 16;39(5):838–47. doi: 10.1093/ndt/gfad239 (PMC11181874; doi:10.1093/ndt/gfad239)

| **Supplementary Table 1. Univariable associations with the dependent variables eGFR baseline, htTKV baseline, eGFR slope and htTKV growth.** | | | | | | | | | | | | | | | |
| --- | --- | --- | --- | --- | --- | --- | --- | --- | --- | --- | --- | --- | --- | --- | --- |
|  | **eGFR baseline** | |  | **htTKV baseline** | |  | | **eGFR slope** | | |  | | **htTKV growth** | | |
|  | **B (95% CI)** | ***P* value** |  | **B (95% CI)** | ***P* value** | |  | | **B (95% CI)** | ***P* value** | |  | | **B (95% CI)** | ***P* value** |
| Log_2_(BHB) | -1.07 (-3.95 to 1.81) | 0.47 |  | 0.961 (0.895 to 1.032) | 0.27 | |  | | 0.35 (0.09 to 0.61) | 0.007 | |  | | 1.000 (0.995 to 1.005) | 0.9 |
| Sex (female) | 11.76 (6.91 to 16.61) | <0.001 |  | 0.637 (0.568 to 0.716) | <0.001 | |  | | 0.67 (0.24 to 1.11) | 0.003 | |  | | 1.004 (0.974 to 0.990) | <0.001 |
| Age (years) | -1.64 (-1.79 to -1.49) | <0.001 |  | 1.016 (1.011 to 1.021) | <0.001 | |  | | -0.01 (-0.02 to 0.01) | 0.50 | |  | | 1.000 (1.000 to 1.000) | 0.9 |
| Log_2_(copeptin) | -14.02 (-15.62 to -12.43) | <0.001 |  | 1.297 (1.242 to 1.355) | <0.001 | |  | | -0.57 (-0.74 to -0.39) | <0.001 | |  | | 1.008 (1.004 to 1.011) | <0.001 |
| SBP (mmHg) | -0.16 (-0.34 to 0.02) | 0.08 |  | 1.006 (1.001 to 1.010) | 0.02 | |  | | -0.04 (-0.05 to -0.02) | <0.001 | |  | | 1.000 (1.000 to 1.000) | 0.10 |
| *PKD2* (ref)* |  |  |  |  |  | |  | |  |  | |  | |  |  |
| *PKD1NT* | 2.64 (-4.05 to 9.33) | 0.44 |  | 0.979 (0.829 to 1.156) | 0.80 | |  | | -1.10 (-1.68 to -0.52) | <0.001 | |  | | 1.003 (0.992 to 1.014) | 0.59 |
| *PKD1T* | 4.46 (-1.67 to 10.6) | 0.15 |  | 1.060 (0.909 to 1.237) | 0.46 | |  | | -1.33 (-1.88 to -0.79) | <0.001 | |  | | 1.007 (0.997 to 1.018) | 0.16 |
| Other/missing | 6.55 (-2.91 to 16.0) | 0.17 |  | 0.851 (0.675 to 1.073) | 0.17 | |  | | -0.13 (-0.96 to 0.70) | 0.75 | |  | | 0.980 (0.965 to 0.994) | 0.008 |
| Estimates, confidence intervals, and *P* values were calculated using linear regression analyses. The dependent variables are eGFR baseline (ml/min/1.73m^2^), log_2_(htTKV) baseline, eGFR slope and log_2_(htTKV) growth. The independent variables are sex, age, log_2_(copeptin), SBP, *PKD1NT, PKD1T*, other/missing mutation. The estimates of htTKV baseline and htTKV growth are back-log transformed, for the categorical variables indicating the fold change in the dependent variable for the specified category compared to the reference category. For the continuous variables, the estimate represents the fold-change per one-unit increase in the continuous variable. When the independent variable is log_2_-transformed, a doubling (one unit increase) of the independent variable corresponds to the fold change according to the estimate.  **PKD* mutation was used as dummy variable with PKD2 as reference group.  *Abbreviations:* B, estimate; CI, confidence interval; N, number; eGFR, estimated glomerular filtration rate; htTKV, height adjusted total kidney volume; SBP, systolic blood pressure; PKD, polycystic kidney disease; NT, non-truncating; T, truncating. | | | | | | | | | | | | | | | |

| **Supplementary Table 2. Longitudinal associations of BHB between eGFR slope using linear mixed model analyses in subgroups** | | | |
| --- | --- | --- | --- |
|  | **BHB est. (95% CI)** | ***P* value** | ***P* value interaction** |
| Men (n=190) | 0.39 (-0.01 to 0.79) | 0.06 | 0.60 |
| Women (n=292) | 0.30 (-0.01 to 0.60) | 0.06 |  |
|  |  |  |  |
| Age < 49 (n=252) | 0.43 (0.06 to 0.80) | 0.02 | 0.59 |
| Age > 49 (n=230) | 0.13 (-0.18 to 0.44) | 0.42 |  |
|  |  |  |  |
| eGFR < 58 (n=240) | 0.26 (-0.02 to 0.54) | 0.07 | 0.77 |
| eGFR > 58 (n=241) | 0.41 (0.04 to 0.78) | 0.03 |  |
|  |  |  |  |
| htTKV < 830 (n=227) | 0.35 (0.01 to 0.68) | 0.04 | 0.56 |
| htTKV > 830 (n=229) | 0.26 (-0.06 to 0.59) | 0.11 |  |
|  |  |  |  |
| Copeptin < 7.8 (n=243) | 0.30 (-0.02 to 0.62) | 0.07 | 0.9 |
| Copeptin > 7.8 (n=239) | 0.29 (-0.06 to 0.64) | 0.11 |  |
|  |  |  |  |
| Total (n=482) | 0.33 (0.09 to 0.57) | 0.008 |  |
| The estimates and *P* values were calculated using linear mixed model analyses. The estimates are the variables and their interaction with time which is the effect of the variables on eGFR (ml/min per 1.73 m^2^) per year per different subgroups. The *P* value for interaction is the *P* value of the variable of the interaction between the subgroup variable, BHB and time. The associations are adjusted for potential confounders: sex, age, copeptin, systolic blood pressure, and *PKD* mutations.  *Abbreviations:* BHB, beta-hydroxybutyrate; Est., estimate; CI, confidence interval; eGFR, estimated glomerular filtration rate; htTKV, height-adjusted total kidney volume; PKD, polycystic kidney disease. | | | |

**Supplementary Figure 1**. Correlation plot between the dependent variables in the multivariable model. Spearman’s correlation was used to determine the correlation coefficient.

*Abbreviations*: SBP, systolic blood pressure; PKD, polycystic kidney disease


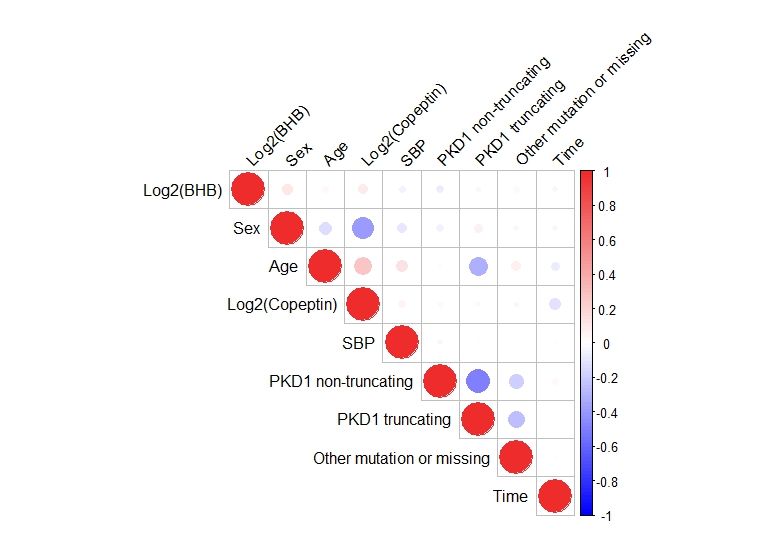


**Supplementary Figure 2**. The line represents the association between glucose and log_2_(BHB), adjusted for sex, age and glucagon. The shaded area is the 95%CI. The dots depict the individual data points.


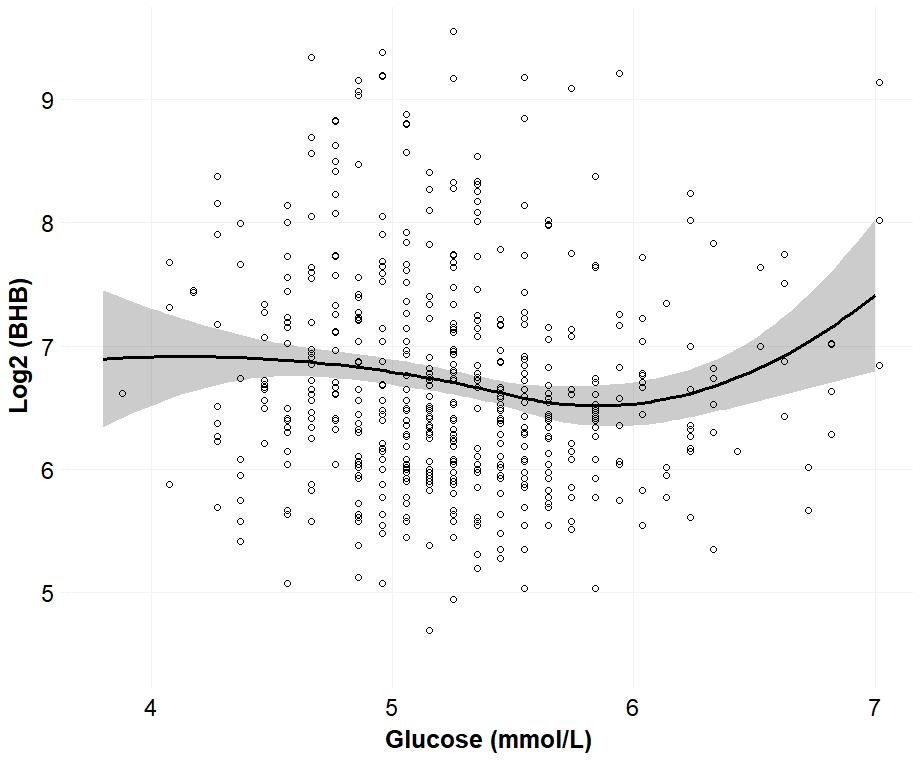

Supplement: gfad239_Supplemental_File [file gfad239_Supplemental_File.docx]
